# A dataset representing the identification of three microalgae species isolated from freshwater areas at Glami Lemi River, Malaysia

**DOI:** 10.1016/j.dib.2022.108761

**Published:** 2022-11-18

**Authors:** Mohd Yusof Hanan, Aznaliza Yahya, Muhamad Zudaidy Jaafar, Md. Ali Amatul-Samahah

**Affiliations:** Freshwater Fisheries Research Division, Fisheries Research Institute Glami Lemi, Jelebu, 71650 Negeri Sembilan, Malaysia

**Keywords:** Aquaculture, Microalgae, 18s rDNA, Phytoplankton, Chlorophyta

## Abstract

Microalgae play an important function in aquatic environments by serving as the energy foundation of the food chain for all aquatic species. Microalgae produce a wide range of bioproducts, including polysaccharides, lipids, pigments, proteins, vitamins, bioactive chemicals, and antioxidants. In this study, a dataset of identification of three microalgae isolated from freshwater riverine areas at Glami Lemi River, Malaysia is presented. The identification data was acquired using molecular identification using the PCR method and morphological observation. The morphological observation of the microalgae isolates, GL01, GL02, and GL03, showed the closest features and characteristics with *Chlorella* sp., *Ankinstrodesmus* sp., and *Tetradesmus* (*Scenedesmus*) sp., respectively. The sequence similarity analysis of partial 18s rDNA gene using BLASTn identified the three microalgae species as *Chlorella sorokiniana* for isolate GL01*, Ankistrodesmus fusiformis* for isolate GL02, and *Tetradesmus obliquus* for isolate GL03. Both morphological observation and molecular identification were in agreement in the determination of the species. This is the first report on the three microalgae species isolated from this area.

**Specifications Table**

**Table:** 

Subject	Greenwater and Environmental Biotechnology
Specific subject area	Microbiology, Genomics, Biotechnology
Type of data	Table, figures
How the data were acquired	The identification data was acquired using molecular identification using the PCR method of partial 18s rDNA gene and morphological observation using a light microscope.
Data format	Raw and analysed
Description of data collection	The microalgae were isolated from the freshwater riverine of Glami Lemi River, Jelebu, Negeri Sembilan, Malaysia. The species microalgae were identified based on morphological observation and molecular identification using 18s rDNA.
Data source location	The microalgae were isolated from the freshwater riverine area of Glami Lemi River, Jelebu, Negeri Sembilan, Malaysia. The data were analysed in the Laboratory of Fish Nutrition in Fisheries Research Institute Glami Lemi, Jelebu, Negeri Sembilan. Latitude and longitude: 3°1′ 23.5416′' N 102°1′ 36.5556′' E
Data accessibility	Data is provided in the article and publicly available at NCBI GenBank https://www.ncbi.nlm.nih.gov/sra/PRJNA871882 https://www.ncbi.nlm.nih.gov/sra/SRX17206935 https://www.ncbi.nlm.nih.gov/sra/SRX17206934 https://www.ncbi.nlm.nih.gov/sra/SRX17206933

## Value of the Data

• The data on the identification of the microalgae add to the growing information database on *Chlorophyta* freshwater species
• The information on the location of the microalgae discovered can help environmental microbiologists better recognize the microorganism's natural habitat.
• The data on morphological observation would enable microbiologists, aquatic biologists and scientists better understand the specific and unique features of each species.
• The data are critical for identifying suitable particular microalgae species for nutritional requirements in the mass production of hygienic live feed, such as *Moina* sp., to be used in the aquaculture sector.

## 1 Objective

**Fig. 1:**
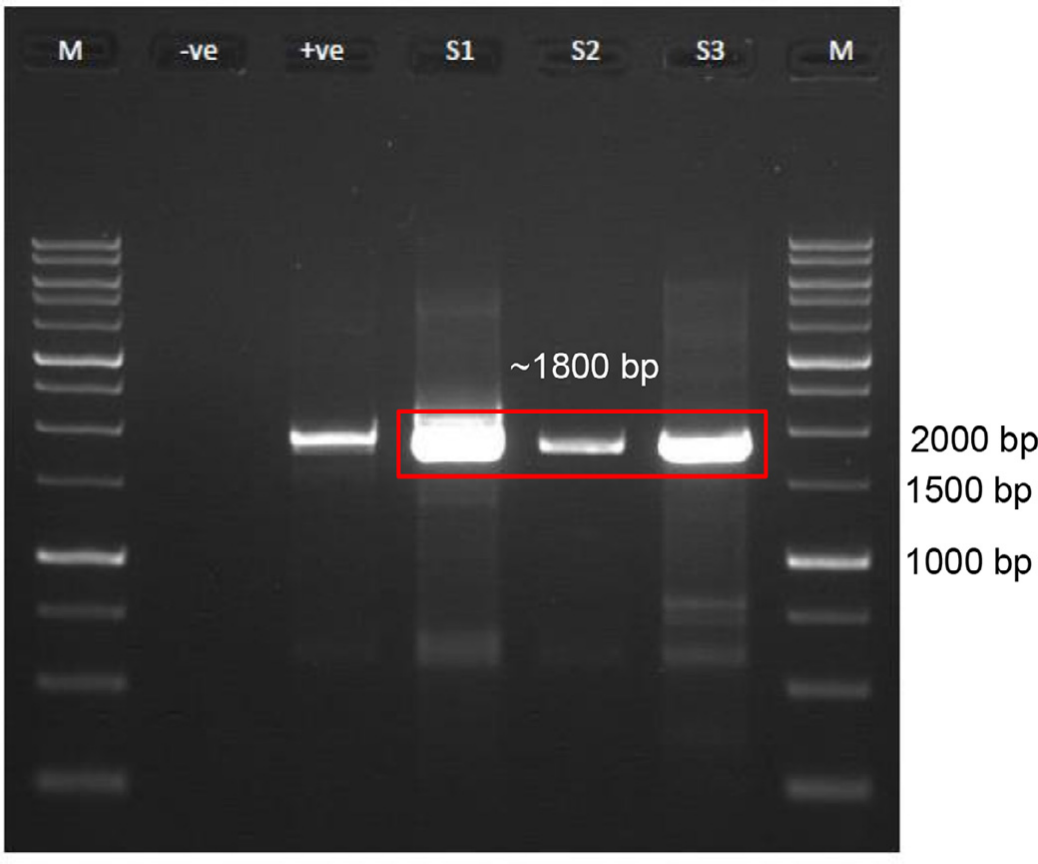
Gel electrophoresis profile of the amplification product of partial 18s rDNA of the three microalgae species isolated from freshwater riverine area of Glami Lemi River, Negeri Sembilan, Malaysia. S1 is sample GL01, S2 is sample GL02, S3 is sample GL03 and M is 1kb DNA Ladder Marker. Negative control (-ve) is a PCR reaction with no template and positive control (+ve) is a PCR reaction from a known organism gDNA.

The objective of this study is to isolate and identify the potential indigenous microalgae species from Malaysian freshwater riverine areas for the cultivation of mass live feed production of *Moina* sp. in the aquaculture industry (Fig. 1).

## 2 Data Description

In this study, the isolates were identified according to their morphological features as observed under a compound microscope and molecular identification. Based on morphological features, Isolate GL01 showed *Chlorella* sp. specific characteristics which were a unicellular cell that is green in color, spherical shape, non-motile, and does not have flagella with their cell size is approximately around 2.5–2.8 µm (Table 1). Isolates GL02 was observed as unicellular, green in color, has elongated cells, appeared in cross-shaped colonies, non-motile, and do not have flagella and the size of the cell width is about 2 µm with a cell length of approximately about 35-45µm. Meanwhile, for isolates GL03, the microalga features were consistent with *Scenedesmus sp*. as the cells appeared unicellular, in coenobia of two cells, or coenobia made up of four or even eight cells, green in color, ovate and have pointy ends, but no spines, non-motile and do not have flagella, and the size of the cell is about 6.0-8.0 µm (Table 1).

**Table 1:** Morphological observation of microalgae isolated from freshwater riverine areas of Glami Lemi River, Negeri Sembilan, Malaysia.

Isolate	Species	Morphological characteristics	Microscopic image
GL01	*Chlorella sorokiniana*	Unicellular, green in color, spherical in shape, non-motile, and does not have flagella, the size of the cell is about 2.5–2.8 µm	
			
GL02	*Ankistrodesmus fusiformis*	Unicellular, green in color, cells elongated, in cross-shaped colonies, non-motile and do not have flagella, size of the cell width about 2 µm, cell length 35-45µm	
			
GL03	*Scenedesmus (Tetradesmus) obliquus*	Unicellular, coenobia of two cells or coenobia made up of four or even eight cells. green in color, ovate and have pointy ends, but no spines, are non-motile and do not have flagella, size of the cell is about 6.0-8.0 µm	
			

**Fig. 2:**
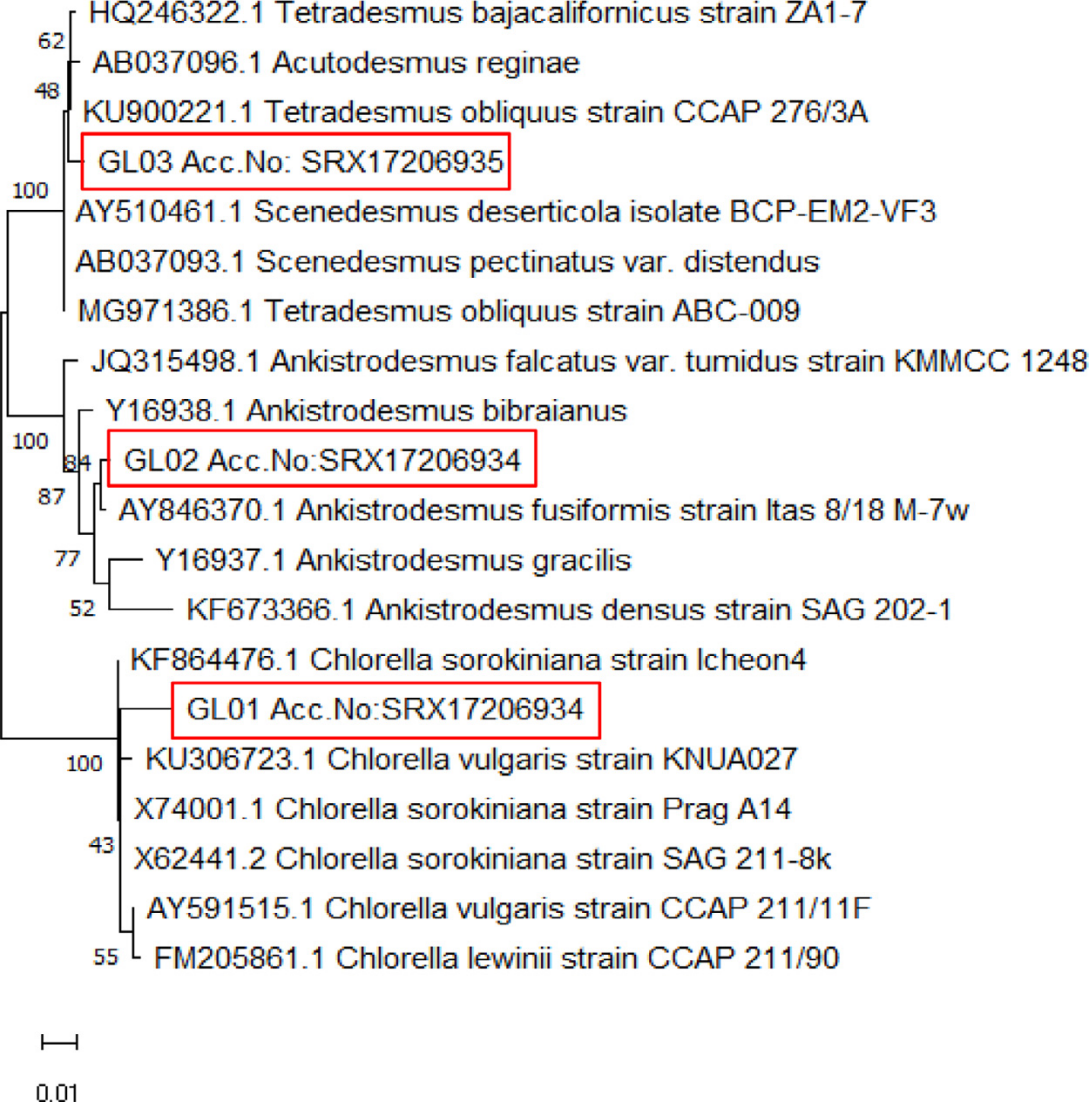
The neighbor-joining (NJ) tree showing the phylogenetic relations among 18S rDNA sequences from microalgae species used in this study and those obtained from the NCBI GenBank database. Bootstrap values 1000.

Based on molecular identification (Table 2) and neighbor-joining (NJ) phylogenetic tree (Fig. 2), isolate GL01 was found to be similar to *Chlorella sorokiniana* (Accession number KF864476.1) with 99.05% of nucleotide similarity and clustered together with *C. sorokinian*a in Fig. 2 with bootstrap values > 90.0%. The strain of GL02 was identified to be similar to *Ankistrodesmus fusiformis* (AY846370.1) with 99.76% of nucleotide similarity and clustered together with *A. fusiformis* with a bootstraps value of 80.0% meanwhile the strain of GL03 were identified as *Scenedesmus (Tetradesmus) obliquus* (Accession number KU900221.1) with 95.81% of nucleotide similarity and clustered together with *T. obliquus* with bootstraps value of 100.0%.

**Table 2:** Molecular identification of the microalgae isolated from freshwater riverine of Sungai Glami Lemi, Negeri Sembilan, Malaysia using sequence similarity search analysis, BLASTn.

Strain	Closest match species	Similarity	Length of sequences (base pairs, bp)	Accession no.
GL01	*Chlorella sorokiniana* (KF864476.1)	99.05%	1786	SRX17206933
GL02	*Ankistrodesmus fusiformis* (AY846370.1)	99.66%	1782	SRX17206934
GL03	*Scenedesmus* (*Tetradesmus*) *obliquus* (KU900221.1)	99.72%	1781	SRX17206935

## 3 Experimental Design, Materials, and Methods

### 3.1 Sampling and isolation

Triplicate 1 L water samples were collected from the freshwater riverine area of Glami Lemi River, Jelebu, Negeri Sembilan, Malaysia (Latitude and longitude: 3°1′ 23.5416′' N 102°1′ 36.5556′' E). The samples were kept in coolers on ice and taken to the laboratory for further analysis. The collected freshwater samples were filtered through glass GF5 grade microfiber filters (pore size 0.7 μm, Chemlab). Then the samples underwent an enrichment step through suspension in liquid Bold's Basal Media (BBM). The tubes were incubated at room temperature (27°C) under continuous cool white fluorescent light for two weeks. The cultures were aerated with approximately 1.5% CO^2^ in the air. After that, the enriched samples were streaked in solidified BBM agar and were incubated under the same conditions described for enrichment for at least four weeks, until individualized green colonies appeared on agar plates. This process was repeated until pure cultures were obtained. The achievement of pure cultures was verified by observation under a light microscope.

### 3.2 Microalgal cultures

The freshwater microalgae (GL01, GL02, and GL03) pure cultures were obtained during the isolation step. These microalgae were previously isolated and cultured from the riverine area of the research institute. The microalgae were cultured in Bold's Basal Medium (BBM). All media were prepared fresh and autoclaved. Starter cultures of 5 mL (previously inoculated from plate agar) were transferred to a 50 mL medium in a 250 mL flask. The tubes were incubated at room temperature (27°C) under continuous cool white fluorescent light. The cultures were aerated with approximately 1.5% CO^2^ in the air.

### 3.3 Morphological observation

Microscopic morphologic analysis was performed using a light microscope at 100x magnification. The features and characteristics of the microalgae isolate GL01, GL02, and GL03, were recorded. Species identification was accomplished by comparing with the original species descriptions available publicly at the AlgaeBase [1].

### 3.4 Species identification of microalgae and phylogenetic analysis

Genomic DNA extraction was done using GeneJET Genomic DNA Purification Kit, Thermo Scientific (USA). The cells were spin-downed by centrifugation at 5000 g, for 10 mins. The supernatant was discarded and the cells were subjected to DNA extraction process. The DNA extraction using GeneJET Genomic DNA Purification Kit was carried out as stated in the manual provided. For molecular identification, the nearly full-length 18s rDNA gene was PCR amplified using the universal eukaryotic primer set (18ScomF1 (forward), 5’-GCTTGTCTCAAAGATTAAGCCATGC-3’; 18ScomR1 (reverse), 5’-CACCTACGGAAACCTTGTTACGAC-3’) [1]. PCR mixtures were 5.0 µl of PCR buffer, 2.0 µl of MgCl_2_ (100 mM), 1.0 µl of dNTPs (10 mM each), 1.0 µl of each forward and reverse primers (20 µM), 0.5 GoTaq Polymerase (5 U µl^−1^) (Promega, USA), 5.0 µL of DNA template (50 ng µl^−1^) and topped up with nuclease-free water (Thermo Fisher Scientific, USA) making up the final volume of 25 µl per reaction. PCR programs were conducted using Eppendorf™ Mastercycler™ Nexus Thermal Cycler (Germany). The PCR amplifications were carried out using the following program, initial denaturation 94˚C for 2 mins then 30 cycles; each cycle, denaturation, 94˚C for 30 secs, annealing, 55˚C for 30 secs, and elongation, 72˚C for 120 secs, then the final elongation step is at 72˚C for 10 mins. The amplified PCR fragments were analyzed by agarose gel electrophoresis (1.0%). Electrophoresis gel was viewed under the Alpha Imager (USA). 1kb Plus DNA Ladder (Thermofisher Scientific) was used as a guide to determine the amplicon size. Next, the purified PCR product was cloned into pJET1.2 and then the positive clone pJET1.2-18SrRNA was sent for sequencing. The sequence similarity search analysis was carried out using the BLASTn program (https://blast.ncbi.nlm.nih.gov/Blast.cgi)) in the nucleotide sequence database at NCBI (http://www.ncbi.nlm.nih.gov/). Phylogenetic analyses were conducted using MEGA 11.0 [2]. Phylogenetic analyses were carried out usneighbor-joiningning method to confirm their taxonomic position and the analysis was carried out using neighbor-joining (Kimura 2-parameter model) of the concatenated phylogenetic tree [3].

## Ethics Statements

Not applicable.

## CRediT authorship contribution statement

**Mohd Yusof Hanan:** Writing – original draft, Writing – review & editing. **Aznaliza Yahya:** Investigation, Writing – review & editing. **Muhamad Zudaidy Jaafar:** Investigation, Writing – review & editing. **Md. Ali Amatul-Samahah:** Investigation, Writing – review & editing, Validation.

## Declaration of Competing Interest

The authors declare that they have no known competing financial interests or personal relationships that could have appeared to influence the work reported in this paper.

## Data Availability

A dataset representing the identification of three microalgae species isolated from freshwater areas at Glami Lemi River, Malaysia (Original data) (NCBI).
